# How Internet use relates to mental health in older adults: findings from the Czech Republic and the Republic of Slovenia

**DOI:** 10.3389/fpubh.2025.1679931

**Published:** 2025-11-26

**Authors:** Monika Klun, Tomas Dosedel, Peter Seljak, Barbara Grintal, Zdenka Milič Kavčič, Jana Goriup, Voyko Kavcic

**Affiliations:** 1 Department of Social Gerontology, Alma Mater Europaea University, Maribor, Slovenia; 2 Department of Sociology, Faculty of Social Studies, Masaryk University, Brno, Czechia

**Keywords:** share, Internet use, older adults, mental health, depression, loneliness, well-being

## Abstract

**Objectives:**

Older adults face challenges in digital engagement, which may be linked to mental health. This study examines associations between Internet use and mental health indicators—depression, loneliness, and well-being—among older adults in the Czech Republic and the Republic of Slovenia, using data from the SHARE Wave 8 survey.

**Methods:**

A sample of 5,201 adults aged 50 + (2,706 Czech, 2,495 Slovenian) was analyzed. Internet use in the past 7 days was the dependent variable. Depression (EURO-D), loneliness (Three-Item Scale), and well-being (CASP-12) served as key independent variables. Binary logistic regression was used, controlling for age, gender, education, and partnership status. To address missing data and enhance analytical robustness, a 1-to-many imputation approach was applied.

**Results:**

Internet use was positively associated with higher well-being and negatively associated with depression and loneliness in both countries. These associations were stronger in the Republic of Slovenia as compared to the Czech Republic. Age and education were the most significant control variables. Women and individuals living alone in the Republic of Slovenia were less likely to use the Internet.

**Conclusion:**

The findings indicate that mental health is significantly associated with Internet use in both countries, though to different degrees, suggesting that national context moderates the relationship between mental health factors and digital engagement. Nonetheless, the binary measure of Internet use represents a limitation, as it does not capture frequency, intensity, or type of online activity. Interventions promoting digital literacy—particularly among older adults with poorer mental health—could enhance digital inclusion and well-being in aging populations.

## 1 Introduction

The European Digital Strategy, alongside related digital inclusion initiatives, underscores the urgent need to increase the proportion of older adults within digitally literate populations. These efforts recognize that digital literacy is essential for ensuring social inclusion, access to digital services, and overall well-being in an increasingly digitalized society (1). The primary goal of these initiatives is to bridge the digital divide by tackling key barriers, such as digital illiteracy, accessibility issues, and technological scepticism among older adults. By doing so, they aim to promote greater participation of aging populations in the digital economy and society.

The Internet and digital technologies offer numerous benefits across various facets of older adults’ lives, from enhancing social connections (1) to improving healthcare access (2) and financial literacy (3). However, overcoming obstacles like digital illiteracy and accessibility challenges is crucial to prevent older adults from being left behind in the digital era. Many older adults remain digitally excluded, which creates a significant gap in both digital access and literacy (4). These challenges are closely related to Digital Divide Theory, which differentiates between the access gap and the usage gap—referring, respectively, to disparities in physical access to technology and in the ability to effectively use it (5, 6). As Xiang and Xing (6) state, the older adults population’s limited access to digital technology, difficulties in using digital devices, and digital poverty in knowledge acquisition can ultimately lead to social differentiation, exclusion, and inequality, reinforcing their marginalization in the digital era.

The demographic shift, namely the growing proportion of adults aged 65 and older in the general population, is not only relevant to the digital divide but also to the factors influencing digital literacy among older adults. With this demographic change, it is reported that more older adults are expected to experience lower subjective well-being (7) and a decline in mental health (8, 9), both of which can negatively impact digital literacy in later life. Thus, we can conclude that the rapid development of Internet technology necessitates a focus not only on cognitive abilities (10–12) but also on mental health in the context of how older adults engage with and are impacted by Internet use. This issue has been often addressed and evaluated with the presence of mental health disorders (e.g., depression and other mood disorders) (9, 10, 13, 14), and by the presence of positive mental health states related topics such as the quality of life (15), and well-being (16–19). These findings underscore the need for a comprehensive approach that integrates both psychosocial and cognitive factors when addressing digital literacy and inclusion among older adults.

When examining digital literacy and digital exclusion among older adults, it is essential to consider the various barriers that hinder Internet use. In addition to challenges related to technological adaptation, older adults often encounter structural, psychological, and socio-economic obstacles that contribute to their digital exclusion. Chang et al. (20) identified several key barriers, including limited access to the Internet, trust-related concerns (e.g., privacy and security issues), and financial constraints. Furthermore, older adults may experience anxiety or fear regarding Internet use, physical limitations associated with psychobiological conditions, language barriers, and apprehension toward adopting information and communication technologies (ICT) and e-services (20–25).

Although older adults are less frequent users of ICT, numerous studies have examined the relationship between characteristics of older people and ICT use. Yu et al. (26) determined that women in their 50s and 60s are more likely to be Internet users compared to their male counterparts. Education significantly influences Internet skills; for instance Augner (10) found indicating that individuals with higher education levels have higher computer skills. Additionally, Hargittai et al. (27) identified a positive correlation between education, income, and Internet skills, while Augner (10) noted that older adults with greater computer skills also experience better mental health. Conversely, social characteristics play a crucial role in Internet skill development; Chang et al. (20) suggest that older adults who are socially isolated or live far from family members tend to have fewer opportunities to enhance their computer skills. In contrast, having a partner has been associated with higher computer skill levels, possibly due to shared learning experiences and emotional support. Additionally, older adults in relationships may experience lower levels of social isolation compared to their single counterparts of the same age (10). This is consistent with Social Capital Theory (28), and aligns with Xiang and Xing (6), who underline the role of Internet use in fostering and maintaining social ties. Social connectedness may thus support the development of digital skills, while online engagement can, in turn, strengthen interpersonal relationships, reduce feelings of isolation, and contribute to greater digital competence and well-being.

Despite all above mentioned digital literacy issues, studies indicate that Internet and ICT use can have significant impacts related to mental health. On the one hand, research (13, 14, 29) indicates that Internet use has positive effects on mental health, especially for older adults who live with their children. It is also linked to a higher level of life satisfaction (16, 19, 30). Lifshitz et al. (17) found that using online functions, including interpersonal communication, information-seeking, and leisure activities, positively correlates with life satisfaction. This notion supports the idea that life satisfaction among older adults is connected to their digital literacy (31). Additionally, Sum et al. (32) found that Internet use enhances older adults’ satisfaction with general health. Moreover, Yang et al. (18), Zhang et al. (1), and Nedeljko (33) reported that Internet use positively influences subjective well-being, with Shapira et al. (16) stating that learning to use computers and the Internet contributes to a sense of empowerment. Other benefits of Internet utilization include increased happiness (32), self-efficacy (31, 34), and autonomy (35). Furthermore, Internet use has been associated with reduced levels of depression (1, 16, 17, 36, 37), anomie (35), and loneliness (16, 32, 38). Zhang et al. (14) found that Internet use positively impacts social interaction and social trust, while Lelkes (30) determined that Internet use decreases social isolation. Sum et al. (32) established a stronger sense of community is linked to older adults’ use of the Internet for information and communication, as well as the frequency and history of their Internet use. Similarly, Gatto and Tak (39) found older adults feel more connected when using email to stay in touch, reconnect with, or establish new friendships. These findings can also be understood through the lens of Social Capital Theory (6, 28), which highlights how digital engagement facilitates the formation and strengthening of social networks, thereby contributing to the development of social capital. In terms of anxiety, Choi and DiNitto (36) report Internet users have a significantly lower level of anxiety symptoms than non-users.

On the other hand, some studies report mixed results. Zhang et al. (1) found that Internet use is not associated with enhanced life satisfaction, and stopping Internet use does not significantly affect life satisfaction or depression. Similarly, Hofer and Hargittai (40) established that general Internet experiences, such as digital literacy, are not linked to anxiety or depression. Hunsaker et al. (41) discovered that belonging to an online community, participating in meaningful online conversations, and having greater involvement in such conversations are all connected to increased anxiety. Furthermore, Hofer and Hargittai (40) found that looking at status updates is adversely correlated with anxiety, and that checking in on someone who has suddenly become absent from an online community can raise anxiety levels. These findings illustrate the complex interplay between digital behaviors and mental health, suggesting that Internet use is not universally beneficial and may carry risks for some individuals.

The reviewed studies on mental health and Internet use present diverse and sometimes conflicting findings, which can be attributed to different methodological approaches [e.g., (18, 35, 37)]. Some studies utilized primary data [e.g., (17, 31, 40)], while others used secondary data from various studies [e.g., (14, 18, 37)] with scarce using data from the Survey of Health, Ageing and Retirement in Europe (SHARE) database [e.g., (10)].

This study presents findings on the associations between Internet use and mental health among older adults in the Czech Republic and the Republic of Slovenia, utilizing data from the SHARE survey. These two nations exhibit both commonalities and differences that provide valuable insights into intercultural and societal dynamics relevant to digital adoption and aging populations. Both countries are experiencing the challenges associated with rapidly aging demographics and have been actively implementing strategies to mitigate the effects of digitalization on older adults. Additionally, they share a history of geopolitical and cultural interactions, having acceded to the European Union in 2004 following the socio-political transformations that resulted from the dissolution of the Eastern Bloc in the late 20th century. Moreover, both nations have encountered comparable difficulties in addressing the repercussions of the COVID-19 pandemic.

As part of this study, it is essential to report digital skills levels in these countries. Specifically, in 2019—the reference year for analyzing the relationship between Internet use and mental health—the proportion of older adults (aged 55–74 years) with low, basic, and above basic digital skills was 65.66% in the Czech Republic and 58.73% in the Republic of Slovenia (42). Despite the observed difference in digital skills between the two nations, we anticipate that the findings concerning the impact of mental health on Internet use will be comparable.

In our current research based on SHARE Wave 8 data for the Czech Republic and the Republic of Slovenia, we found that lower cognitive abilities significantly reduce ICT use among older adults, even after excluding individuals with probable cognitive impairments (43). This association remained robust even after controlling for key demographic and socioeconomic factors, including gender, age, education, and living conditions.

Building upon prior analysis, the present study investigates the association between mental health and Internet use among older adults. Specifically, it examines how mental health indicators—depressive symptoms, loneliness, and life satisfaction—are associated with digital behavior in later life. Drawing on data from Wave 8 of the SHARE project, the study focuses on older adults in the Czech Republic and the Republic of Slovenia, two countries with comparable demographic trends and digital inclusion challenges. The core aim of this study is to answer the following research question: How does the mental health of older adults influence their use of digital services?

We hypothesize a significant relationship between Internet use and mental health, specifically:

*H1.1*: Older adults with lower self-reported depressive symptoms are more likely to use the Internet than those who self-report higher levels of depressive symptoms.

*H1.2*: Older adults with lower self-reported loneliness are more likely to use the Internet than those with higher levels of loneliness.

*H1.3*: Older adults with higher self-reported well-being are more likely to use the Internet compared to those reporting lower well-being.

Additionally, we hypothesize that the associations between Internet use and mental health among older adults do not vary across national contexts:

*H2*: There are no differences in associations between Internet use among older adults and their mental health in the Czech Republic and the Republic of Slovenia.

## 2 Research methods

To investigate the association between mental health and Internet use in older adults, we analyzed data from Wave 8 of the Survey of Health, Ageing and Retirement in Europe (SHARE), a longitudinal panel survey covering individuals aged 50 and above. Wave 8, conducted at the onset of the COVID–19 pandemic, was selected as it includes relevant indicators on digital behavior and mental health (44, 45).

From the wide array of available modules, three were selected for the purpose of this study. The Information Technology (IT) module provided the dependent variable, measuring Internet use. The Mental Health (MH) and Activities (AC) modules contributed the independent variables, capturing respondents’ self-reported levels of depressive symptoms, loneliness, and well-being.

To address the issue of missing data and enhance analytical robustness, a 1-to-many imputation approach was applied using prepared gv_imputations module. This method allows each original respondent to be represented by multiple imputed cases while maintaining the representativeness and statistical integrity of the dataset. According to the documentation for the SHARE (41) and the methodological article (46), the hot-deck technique was used to impute missing non-monetary values, utilizing the socio-demographic characteristics of the respondents. Five independent datasets were created, which were combined in further iterations until the values converged and became independent of the original settings (46). However, we obtained a ready-made file for imputations along with other SHARE modules and limited ourselves to linking it with other modules.

After filtering for participants aged 50 and above residing in the Czech Republic and the Republic of Slovenia, the final analytical sample included 23,790 cases (12,160 from the Czech Republic and 11,630 from Slovenia), based on an initial unweighted sample of 5,201 respondents (2,706 from the Czech Republic and 2,495 from Slovenia).

### 2.1 Dependent variables

The central outcome of interest in this study is Internet use among older adults, which serves as the dependent variable. This variable was derived from the SHARE Wave 8 question: *“During the past 7 days, have you used the Internet for e-mailing, searching for information, making purchases, or for any other purpose at least once?”*

This measure was selected because it captures recent and general digital activity, and unlike other available indicators, it is consistently included in Wave 8 across countries. Its focus on weekly Internet use ensures that the variable reflects current engagement rather than outdated or infrequent usage. Responses to this question were binary-coded to enable a clear distinction between Internet users and non-users:

- 0 = No, the respondent did not use the Internet in the past 7 days
- 1 = Yes, the respondent used the Internet in the past 7 days

This dichotomous classification is appropriate for the use of binary logistic regression, which enables the estimation of the likelihood of Internet use as a function of key mental health indicators—namely depressive symptoms, loneliness, and life satisfaction. The coding scheme also ensures compatibility with multivariate statistical models used to test the proposed hypotheses.

### 2.2 Independent variables

To examine the association between mental health and Internet use among older adults, three independent variables were included: depression, loneliness, and well-being (47). Depression was measured using the EURO-D scale, a validated instrument designed for cross-national comparisons of late-life depression (48). In SHARE, this scale is available as a generated composite variable within the Mental Health (MH) module. It consists of 12 binary items (yes/no), assessing symptoms such as sadness, pessimism, sleep disturbance, and lack of interest. Scores range from 0 to 12, where higher values indicate more pronounced depressive symptoms. A threshold score of **≥**4 is used to classify respondents as likely depressed, whereas scores **<**4 suggest the absence of significant depressive symptoms. This cut-off facilitates international comparability and enables an assessment of how depressive states influence digital service use among older adults.

Loneliness was measured using the Three-Item Loneliness Scale developed by Hughes et al. (49). This short-form scale captures emotional and social dimensions of loneliness via three questions related to the frequency of feeling a lack of companionship, being left out, and feeling isolated. Responses are recorded on a three-point Likert scale (1 = Hardly ever or never, 2 = Some of the time, 3 = Often), producing a total score ranging from 3 to 9. 3 represents the lowest level of loneliness and 9 represents the highest level of loneliness. The summed score was derived from items in the MH module and offers a concise yet reliable measure of subjective social disconnection among older adults.

Well-being was operationalized using the CASP-12 scale (Control, Autonomy, Self-Realization, and Pleasure), included in the Activities (AC) module as a generated variable (50). This instrument assesses quality of life in older age by measuring four psychosocial domains: (1) Control—the ability to actively shape one’s environment, (2) Autonomy—the sense of independence and freedom from external constraints, (3) Self-realization—the pursuit of personal growth and achievement, and (4) Pleasure—enjoyment and fulfilment in everyday life. The scale comprises 12 items, each rated on a four-point Likert scale (1 = Never, 2 = Rarely, 3 = Sometimes, 4 = Often), yielding scores from 12 (lowest well-being) to 48 (highest well-being). Higher CASP-12 scores indicate greater life satisfaction and emotional well-being, offering a holistic perspective on the psychological resources available to support digital engagement. By including these three distinct but complementary indicators, the study aims to capture a comprehensive picture of mental health and its potential role in shaping patterns of Internet use among older adults.

### 2.3 Control variables

In order to isolate the effects of mental health indicators on Internet use, several control variables were included in the analysis: age, gender, years of education, and cohabitation with a partner. These variables were selected based on prior research indicating their relevance for both digital engagement and psychological well-being among older adults.

Age was treated as a continuous variable, representing the respondents’ age at the time of the survey. In the Czech sample, participants ranged from 51 to 96 years (*M* = 72.24), while the Slovenian sample included respondents aged 50–100 years (*M* = 71.51). Although the Slovenian sample was slightly younger on average, it encompassed a broader age range.

Gender was included as a dichotomous variable (1 = male, 2 = female). The gender composition was similar across both samples, with females comprising 60.9% of the Czech respondents and 59.8% of the Slovenian sample.

Educational attainment was measured as the total number of years spent in formal education. The Czech respondents reported a higher average educational level (*M* = 12.48 years) compared to the Slovenian respondents (*M* = 10.75 years).

Living with a partner was also treated as a binary variable (0 = no residential partner, 1 = living with a partner). In the Slovenian sample, 27.6% of respondents lived alone (i.e., were single, widowed, divorced, or separated), while this proportion was higher in the Czech sample (37.9%). Descriptive statistics for all variables, including control variables, are presented in Table 1, along with *p*-values from independent samples t-tests. These tests assess the statistical significance of cross-country differences, ensuring that demographic and socio-structural disparities are accounted for in the interpretation of regression results.

**Table 1:** Descriptive statistics.

Variable	The Czech Republic				The Republic of Slovenia				CZ vs. SI
	Min	Max	Mean	SD	Min	Max	Mean	SD	*t*-test *p*=
Internet usage	0	1	0.561	0.496	0	1	0.463	0.499	0.000
Age	51	96	72.244	7.738	50	100	71.513	8.877	0.000
Gender	1	2	1.609	0.488	1	2	1.598	0.490	0.084
Years in education	1	25	12.482	3.123	0	24	10.751	3.361	0.000
Living with partner	0	1	0.621	0.485	0	1	0.724	0.447	0.000
Depression	0	11	2.074	1.989	0	12	2.230	2.096	0.000
Loneliness	3	9	4.162	1.354	3	9	3.781	1.211	0.000
Well-being	18	48	36.527	5.486	18	48	38.911	5.760	0.000

Source: Share Wave 8, *N* = 23,790 (12,160 for the Czech Republic, 11,630 for the Republic of Slovenia). Depression measured using EURO-D scale; Loneliness measured using Three-Item Loneliness Scale; Well-being measured using CASP-12 scale. CZ, the Czech Republic; SI, the Republic of Slovenia.

### 2.4 Analytical procedures

The statistical analyses were conducted using Stata 16 IC and proceeded in three sequential steps, aimed at building robust binary logistic regression models for each country. In the first step, all continuous variables were standardized to ensure comparability of regression coefficients and to facilitate interpretation. Standardization was performed using *z*-scores, transforming the variables such that their mean equals 0 and standard deviation equals 1. This procedure is particularly useful when variables are measured on different scales. In the second step, we assessed the bivariate associations between the dependent variable (Internet use), the independent variables (depression, loneliness, well-being), and the control variables (age, gender, education, partnership status) using Pearson’s correlation coefficients. This preliminary analysis provided insight into the strength and direction of relationships among variables prior to multivariate modeling. In the third step, we estimated a series of binary logistic regression models, conducted separately for the Czech Republic and the Republic of Slovenia. These models examined the likelihood of Internet use as a function of mental health indicators and control variables. For easier interpretability, we reported odds ratios (ORs) instead of raw logistic coefficients. Categorical variables such as gender and partnership status were included in the models as dummy variables, while continuous variables (age, years of education, and the three mental health scales) were entered in their standardized form. This modeling strategy allows for clearer comparisons across variables and between countries.

## 3 Results

The correlation analysis revealed statistically significant associations between Internet use and the examined variables in both the Czech Republic and the Republic of Slovenia. Among all observed variables, age exhibits the strongest and most consistent negative association with Internet use. Specifically, older respondents are less likely to use the Internet (*r* = −0.366 for the Czech Republic; *r* = −0.449 for the Republic of Slovenia). In contrast, years of education are positively associated with Internet use, indicating that higher educational attainment increases the likelihood of Internet use (*r* = 0.316 for the Czech Republic; *r* = 0.367 for the Republic of Slovenia). Furthermore, the presence of a residential partner also increases the probability of Internet use (*r* = 0.114 in the Czech Republic; *r* = 0.189 in Slovenia), while being female slightly decreases the probability of Internet use in both countries (*r* = −0.048 for the Czech Republic; *r* = −0.061 for the Republic of Slovenia). Regarding the independent variables representing mental health, the findings show that higher levels of depression and loneliness are associated with lower Internet use, while higher well-being is positively associated with Internet use. Specifically, depression is negatively associated with Internet use (*r* = −0.137 in the Czech Republic; *r* = −0.229 in the Republic of Slovenia), as does loneliness (*r* = −0.144 in the Czech Republic and *r* = −0.198 in the Republic of Slovenia). Well-being, in turn, shows a positive association (*r* = 0.166 in the Czech Republic; *r* = 0.302 in the Republic of Slovenia) (Table 2).

**Table 2:** Correlation matrix.

Variable	The Czech Republic	The Republic of Slovenia
	Internet use (*r*)	Internet use (*r*)
Age	−0.366	−0.449
Gender	−0.048	−0.061
Years in education	0.316	0.367
Living with a partner	0.114	0.189
Depression	−0.137	−0.229
Loneliness	−0.144	−0.198
Well-being	0.166	0.302

Source: Share Wave 8, *N* = 23,790 (12,160 for the Czech Republic, 11,630 for the Republic of Slovenia); all the correlation coefficients are statistically significant at *p* < 0.05. Depression measured using EURO-D scale; Loneliness measured using Three-Item Loneliness Scale; Well-being measured using CASP-12 scale.

The analysis also revealed significant associations between age and mental health indicators. In both countries, older respondents tend to report more depressive symptoms and greater loneliness, along with lower well-being. The association between age and depression is *r* = 0.091 in the Czech Republic and *r* = 0.180 in the Republic of Slovenia, while the association between age and loneliness is *r* = 0.124 and *r* = 0.185, respectively. Inversely, age is negatively associated with well-being (*r* = −0.135 in the Czech Republic; *r* = −0.259 in the Republic of Slovenia).

To further explore these relationships, we constructed a series of binary logistic regression models. The first model (M1), which included only the three mental health scales, explained 2.6% of the variance in Internet use for the Czech Republic and 7.8% for the Republic of Slovenia (Pseudo *R*^2^). In the second model (M2), we added age to the regression. The inclusion of age markedly improved the explanatory power of the model, raising the Pseudo *R*^2^ to 11.9% for the Czech Republic and 20.4% for the Republic of Slovenia. These results confirm the strong and consistent influence of age on Internet use. The final model (M3) incorporated all control variables—age, gender, years of education, and presence of a residential partner—alongside the mental health indicators. This comprehensive model further improved the explanatory power, with Pseudo *R*^2^ values reaching 19.4% in the Czech Republic and 28.8% in the Republic of Slovenia (Table 3).

**Table 3:** Determinants of Internet use in last 7 days (binary logistic regression, odds ratios).

Variables	The Czech Republic					
	M1		M2		M3	
	Odds ratio	*p* > *z*	Odds ratio	*p* > *z*	Odds ratio	*p* > *z*
Depression	0.887	0.000	0.884	0.000	0.907	0.000
Loneliness	0.871	0.000	0.907	0.000	0.923	0.002
Well-being	1.250	0.000	1.196	0.000	1.173	0.000
Age			0.447	0.000	0.421	0.000
Gender (male = ref)					0.975	0.578
Years in education					2.187	0.000
Partnership status (no partner = ref)					1.022	0.646
Constant	1.284	0.000	1.316	0.000	1.361	0.000
*N*	12,160		12,160		12,160	
Pseudo *R*^2^	0.026		0.119		0.194	

Variables	The Republic of Slovenia					
	M1		M2		M3	
	Odds ratio	*p* > *z*	Odds ratio	*p* > *z*	Odds ratio	*p* > *z*
Depression	0.815	0.000	0.818	0.000	0.824	0.000
Loneliness	0.886	0.000	0.887	0.000	0.903	0.000
Well-being	1.672	0.000	1.499	0.000	1.331	0.000
Age			0.352	0.000	0.317	0.000
Gender (male = ref)					0.874	0.005
Years in education					2.430	0.000
Partnership status (no partner = ref)					1.305	0.000
Constant	0.828	0.000	0.770	0.000	0.650	0.000
*N*	11,630		11.630		11,630	
Pseudo *R*^2^	0.078		0.204		0.288	

Source: Share Wave 8. Depression measured using EURO-D scale; Loneliness measured using Three-Item Loneliness Scale; Well-being measured using CASP-12 scale.

According to the final models, all three mental health variables significantly affect the probability of Internet use. The increase on the depression scale is associated with a decrease in the odds of Internet use in the Czech Republic (9% per one standard deviation) and in the Republic of Slovenia (18% per one standard deviation). Similarly, each increase of standard deviation of the loneliness scale reduces the odds by 8 and 10%, respectively. On the other hand, well-being exerts a positive influence: each additional point on the CASP–12 scale increases the likelihood of Internet use by 17% in the Czech Republic and 33% in the Republic of Slovenia.

Among the control variables, age and education had the strongest effects. Each additional standard deviation of age reduced the odds of Internet use by 58% in the Czech Republic and by 68% in the Republic of Slovenia. Conversely, each additional standard deviation spent in education increased the odds of Internet use by 119% for Czech respondents and 143% for Slovenian respondents.

Gender and partnership status showed weaker and more context-dependent effects. In the Czech Republic, women were 2.5% less likely to use the Internet, though this difference was not statistically significant. In the Republic of Slovenia, however, women were 13% less likely to be Internet users, and this difference was significant. Finally, living with a partner increased the odds of Internet use by 30% in the Republic of Slovenia but had no significant effect in the Czech Republic.

**Figure 1:**
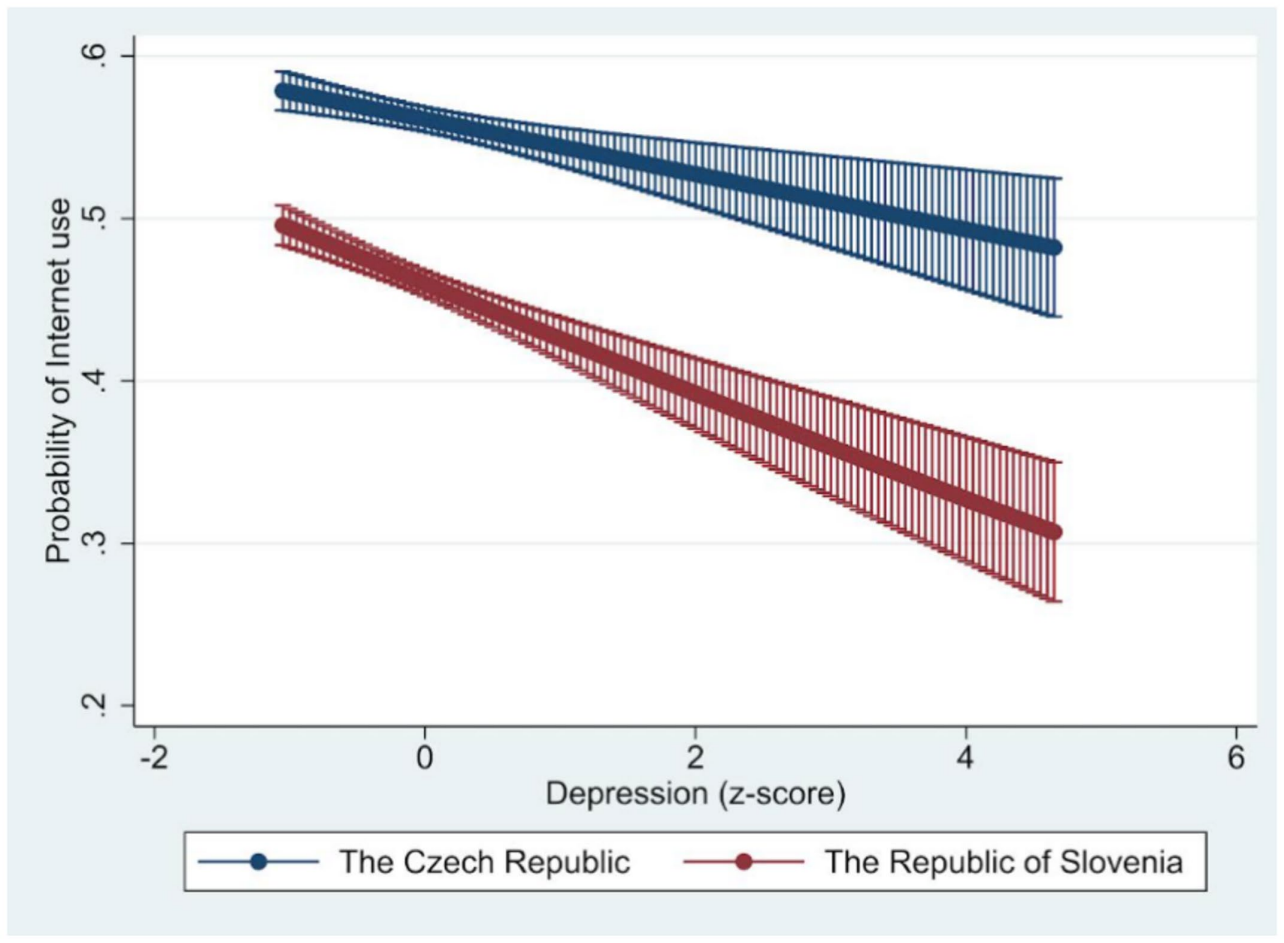
Marginal effect of depression on the probability of Internet use. Source: Share Wave 8. Depression measured using EURO-D scale; Marginal effects predicted from model M4, 95% CI lines.

**Figure 2:**
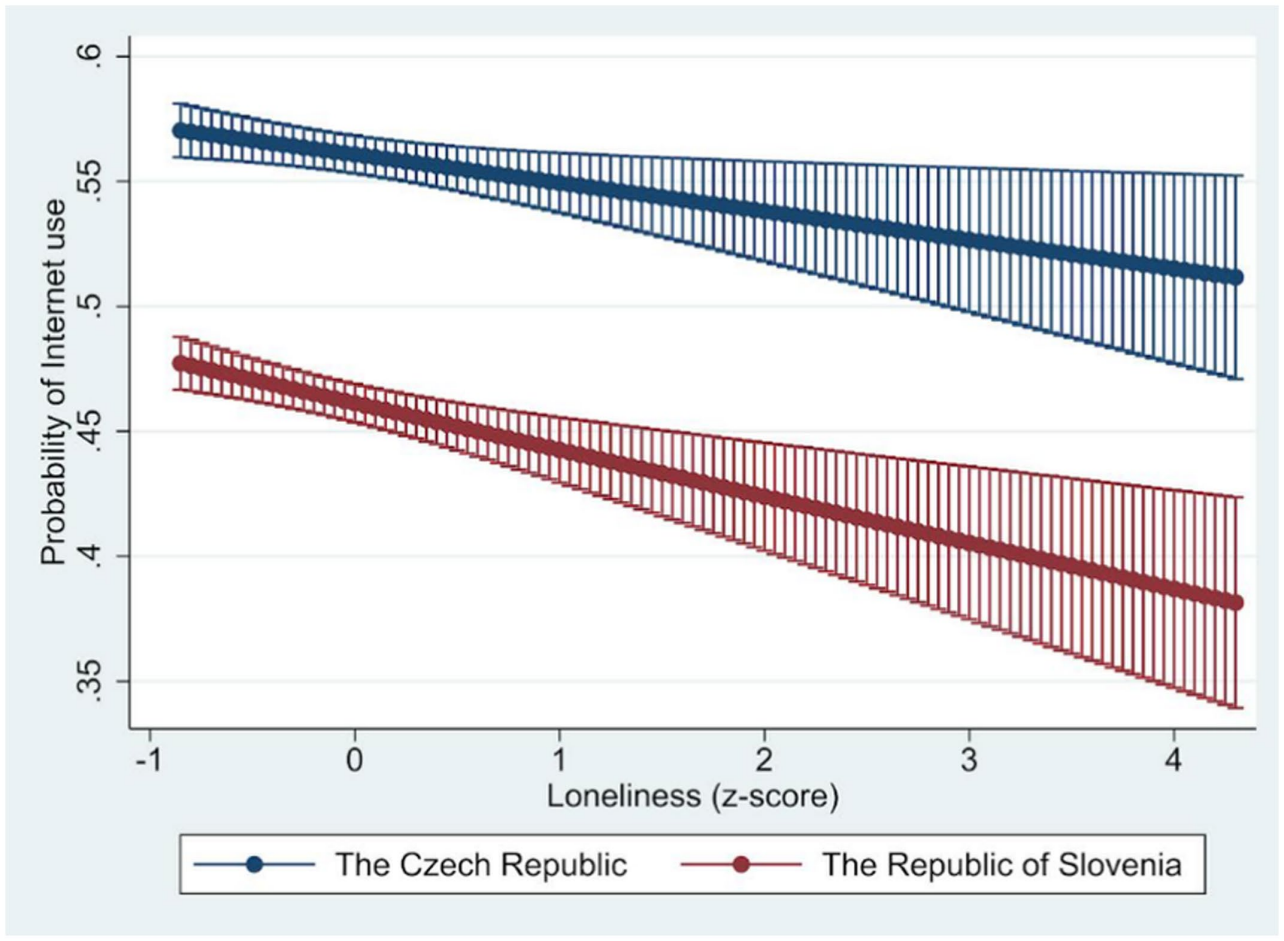
Marginal effect of loneliness on the probability of Internet use. Source: Share Wave 8. Loneliness measured using Three-Item Loneliness Scale; Marginal effects predicted from model M4, 95% CI lines.

**Figure 3:**
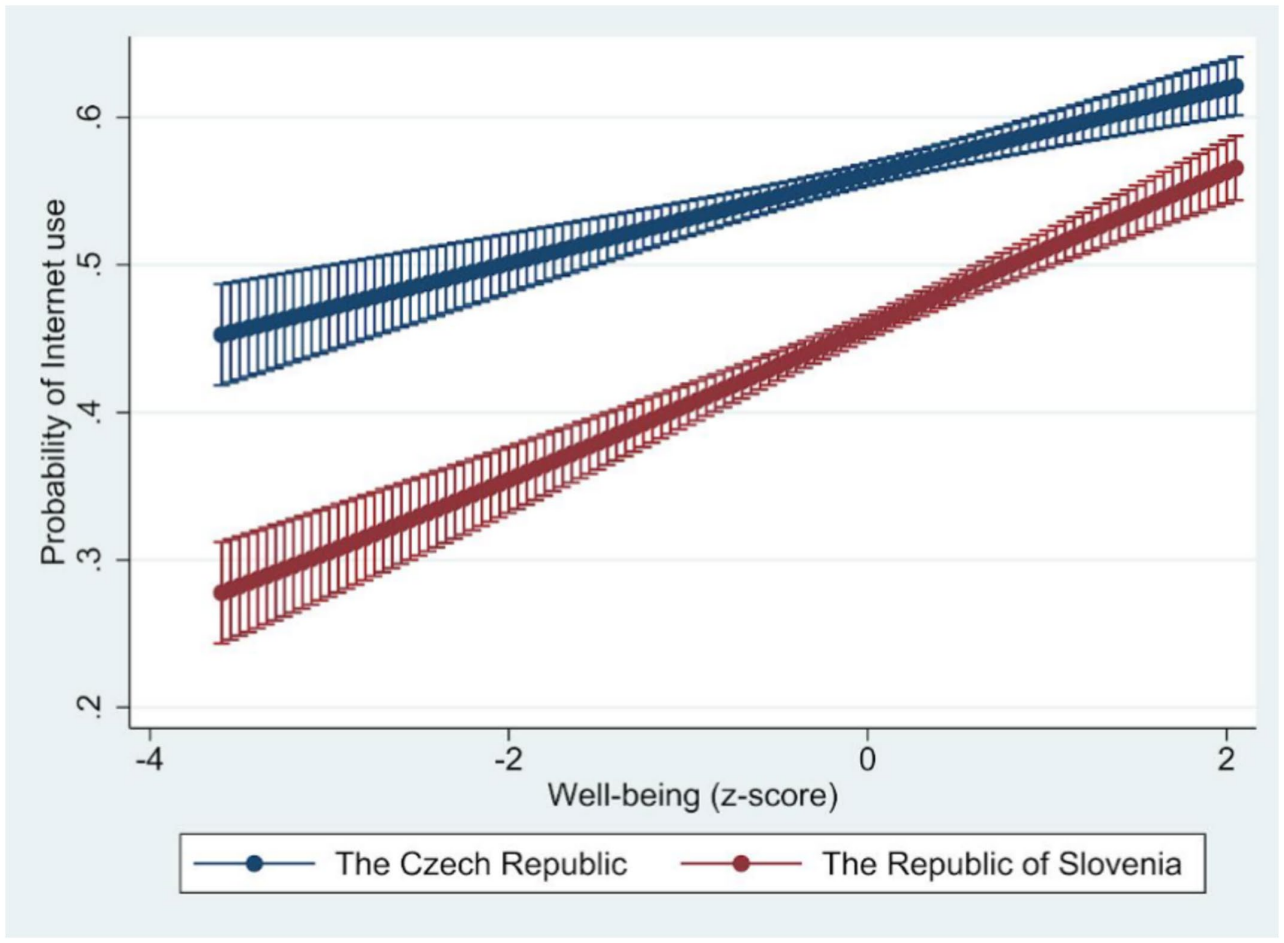
Marginal effect of well-being on the probability of Internet use. Source: Share Wave 8. Well-being measured using CASP-12 scale; Marginal effects predicted from model M4, 95% CI lines.

We additionally estimated a joint model M4 (Table 4) for both countries, which is based on the M3 models, but where the main explanatory variables (depression, loneliness, and well-being) were present both separately and in interaction for individual countries. As interaction terms in non-linear models such as logistic regression are not directly interpretable from model coefficients, the results are presented in the form of marginal effects, which allow for a more meaningful examination of the conditional relationships between predictors and Internet use (see Figures 1–3). For depression, an increase leads to a lower odds ratio of the Internet use. The effect is lower, but the decline is faster for the Republic of Slovenia. An increase in loneliness also leads to a lower odds ratio of the Internet use, and in this case, the effect is lower for the Republic of Slovenia than for the Czech Republic. For well-being, on the other hand, the higher the values on this scale, the more the odds ratio of the Internet use increases. The effect is again weaker for the Republic of Slovenia, but the effects gradually converge in both countries at higher values.

**Table 4:** Joint model.

Variables	M4	
	Odds ratio	*p* > *z*
Depression	0.911	0.000
Loneliness	0.939	0.015
Well-being	1.178	0.000
Age	0.373	0.000
Gender (male = ref)	0.930	0.030
Years in education	2.286	0.000
Partnership status (no partner = ref)	1.128	0.000
Country (CZ = ref)	0.541	0.000
Country * Depression	0.903	0.007
Country * Loneliness	0.956	0.235
Country * Well-being	1.140	0.001
Constant	1.327	0.000
*N*	23.790	
Nagelkerke Pseudo *R*^2^	0.381	

Source: Share Wave 8. Depression measured using EURO-D scale; Loneliness measured using Three-Item Loneliness Scale; Well-being measured using CASP-12 scale. CZ, the Czech Republic.

## 4 Discussion

This study examined the relationship between mental health indicators—specifically depressive symptoms, loneliness, and subjective well-being and Internet use among older adults, using data from the SHARE survey for the Czech Republic and the Republic of Slovenia. The findings indicate that mental health indicators are significantly associated with Internet use, providing relevant evidence for evaluating the proposed hypotheses in two distinct national contexts. By providing data from two countries, our study addresses the noted lack of cross-national research on this topic in older populations (51) and offers new insights into how these associations might vary by context.

Older adults with lower self-reported depressive symptoms are more likely to use the Internet than those reporting higher levels of depressive symptoms, thereby supporting Hypothesis H1.1. This finding is consistent with prior studies that highlight a negative association between depression and Internet use (1, 16, 17, 36, 37). These studies emphasize the potential of digital technologies to support mental health by enabling access to information, cognitive stimulation, and social interaction. However, our results differ from those of Hofer and Hargittai (40), who found no significant relationship between Internet use and depression. This divergence can be attributed to methodological differences, sample characteristics, or contextual factors such as digital infrastructure, social support systems, or national digital literacy initiatives. Further comparative studies are needed to better understand these contextual moderating factors.

Older adults with lower self-reported loneliness and higher well-being are more likely to use the Internet, confirming Hypotheses H1.2 and H1.3. These findings are in line with previous research showing that Internet use is associated with reduced feelings of loneliness (16, 32, 38) and enhanced well-being (1). As Sum et al. (32) explain, older adults may compensate for feelings of social isolation by turning to digital communication tools, which offer opportunities for connection, entertainment, and engagement with the broader world. This interpretation is further supported by Xiang and Xing (6), who emphasize that digital participation can help older adults foster social connectedness and mitigate exclusion, thereby contributing to psychological well-being in later life.

To test Hypothesis 2, which assumed no cross-national differences, we estimated a pooled model with interaction terms. Contrary to H2, the results revealed significant differences in magnitude between the Czech Republic and the Republic of Slovenia, though the direction of effects was consistent in both countries. Better well-being increased, and higher depression and loneliness decreased, the likelihood of Internet use, but these associations were systematically stronger in the Republic of Slovenia. This suggests that mental health disparities play a more decisive role in shaping digital engagement among older Slovenians than among their Czech counterparts. The stronger associations in the Republic of Slovenia may reflect cultural, structural, and policy-related factors. For example, gender differences and partner effects were significant in the Repubic of Slovenia but not in the Czech Republic, indicating that household support and gendered barriers may shape digital engagement differently. The Republic Slovenia’s more recent digital inclusion initiatives could also amplify disparities: older adults with good mental health may be better positioned to take advantage of these programs, while those with poorer mental health may remain excluded. In contrast, Czech older adults may experience broader Internet penetration, making mental health a less decisive factor in distinguishing users from non-users.

Beyond mental health, demographic and socio-demographic variables also influenced Internet use differently across countries. Gender was not significant in the Czech Republic, but in the Republic of Slovenia women were significantly less likely to use the Internet than men, pointing to gendered barriers to digital inclusion (see Table 3). Living with a partner was positively associated with Internet use in the Republic of Slovenia, but not in the Czech Republic, suggesting that cohabitation may facilitate digital engagement through shared resources and informal support (see Table 3). Interestingly, individuals living alone were not more inclined to use the Internet to compensate for reduced in-person contact, contrary to expectations. These differences may reflect structural inequalities consistent with Digital Divide Theory (5, 6), which emphasizes that both access to technology and the ability to use it effectively are shaped by socio-demographic and cultural factors.

Taken together, these findings underscore that digital inclusion is not only a technical matter of access and skills, but also deeply intertwined with mental health, gender, and household structures. In the Republic of Slovenia, where mental health and socio-demographic disparities are more pronounced, interventions may need to target older adults with depression or loneliness, while also addressing gendered barriers and involving family members in training. In the Czech Republic, where the gradients are weaker, broad digital access programs remain important but should still account for psychosocial influences. More broadly, our study highlights the importance of national context in shaping digital behavior and calls for comparative approaches that integrate cultural, structural, and policy-related perspectives into both theory and practice.

These findings have several practical implications. They highlight the importance of integrating mental health considerations into digital literacy and inclusion initiatives for older adults. Tailored interventions—such as community-based workshops or digital skills programs—could target individuals with limited prior exposure to digital tools and those facing obstacles that contribute to their digital exclusion (20–22, 24). Given that living with a partner is positively associated with Internet use, such programs may also benefit from involving family members, caregivers, or volunteers. From a policy perspective, these findings underline the need to develop socially inclusive strategies that address both digital and mental health disparities among aging populations. National digital inclusion strategies should explicitly target gender and social inequalities influencing Internet use. Policies that focus on reducing gender-specific barriers and consider social factors such as living arrangements can better support vulnerable groups. Integrating mental health support with digital literacy initiatives and fostering collaboration between government, community organizations, and healthcare providers could contribute to reducing the digital divide and improving well-being among older adults.

Further research is needed to clarify the directionality of the observed associations. While our results show significant associations between mental health and Internet use, the cross-sectional design of this study limits the ability to draw causal conclusions. Future research should utilize longitudinal and mixed-method approaches to disentangle the directionality of effects and explore mediating variables such as digital self-efficacy, social support, and ICT accessibility. Including perspectives from older adults’ family members and national-level contextual factors may also provide deeper insight into the digital experiences of older adults across countries.

This study has additional methodological limitations. The measure of Internet use relies on a single-item indicator, which does not capture important dimensions such as frequency, type of use (communication, information, leisure), or intensity (52, 53). Although previously validated (54), this limited measurement constrains understanding of how different patterns of Internet engagement relate to mental health outcomes. Therefore, future studies should incorporate more nuanced and comprehensive measures of Internet use to better capture its complexity. Moreover, the measurement of mental health was limited to selected indicators and may not capture the full complexity of psychological well-being. Other limitations include reliance on self-reported data, which may be subject to recall bias or social desirability bias, especially concerning mental health or ICT use. Incorporating additional control variables, such as actual Internet access, digital skills, attitudes toward ICT, or problematic use (55), would also provide a more nuanced understanding of the relationship between Internet use and mental health. Finally, the application of theoretical frameworks like the I-PACE model (56, 57) could further illuminate the cognitive and emotional mechanisms underlying patterns of Internet use and maladaptive digital behaviors in older adults.

Our findings underscore the importance of addressing mental health disparities in efforts to promote digital inclusion among older adults. While better mental health was consistently associated with higher Internet use across both countries, the strength of these associations differed: effects of depression, loneliness, and well-being were systematically stronger in the Republic of Slovenia than in the Czech Republic, with the gap in well-being narrowing only at higher levels. This suggests that national context moderates the relationship between psychosocial factors and digital engagement. Future studies should therefore continue to explore the multifaceted interplay between mental health and technology use in later life, with particular attention to how policy environments, cultural attitudes, and intergenerational support systems shape these dynamics across different societies.

## Data Availability

Publicly available datasets were analyzed in this study. This data can be found here: doi 10.6103/SHARE.w8ca.800.
